# Supplementary material for: Oxidative Distress Induces Wnt/β-Catenin Pathway Modulation in Colorectal Cancer Cells: Perspectives on APC Retained Functions
Source: Cancers (Basel). 2021 Nov 30;13(23):6045. doi: 10.3390/cancers13236045 (PMC8656656; doi:10.3390/cancers13236045)
Supplement: Supplementary file 1 [file cancers-13-06045-s001.zip › cancers-1465288-supplementary.pdf]

Supplementary Materials: Oxidative Distress Induces Wnt/β-Catenin Pathway Modulation in Colorectal Cancer Cells: Perspectives on APC Retained Functions

Teresa Catalano, Emira D'Amico, Carmelo Moscatello, Maria Carmela Di Marcantonio, Alessio Ferrone, Giuseppina Bologna, Federico Selvaggi, Paola Lanuti, Roberto Cotellese, Maria Cristina Curia, Rossano Lattanzio and Gitana Maria Aceto

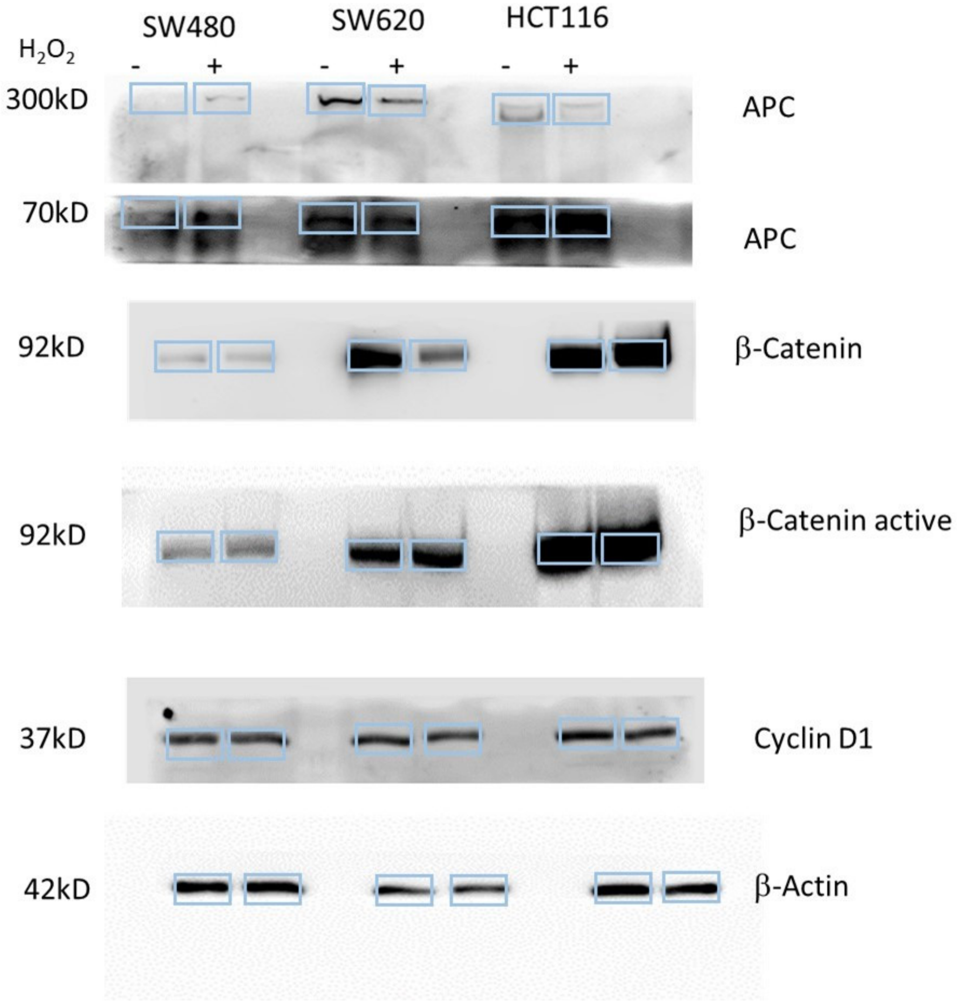

Figure S1. Immunoreactive bands for each protein marker analyzed and its relative molecular weight in kD. The areas delimited by the boxes in light blue color, are indicative of what was assessed in densitometry.

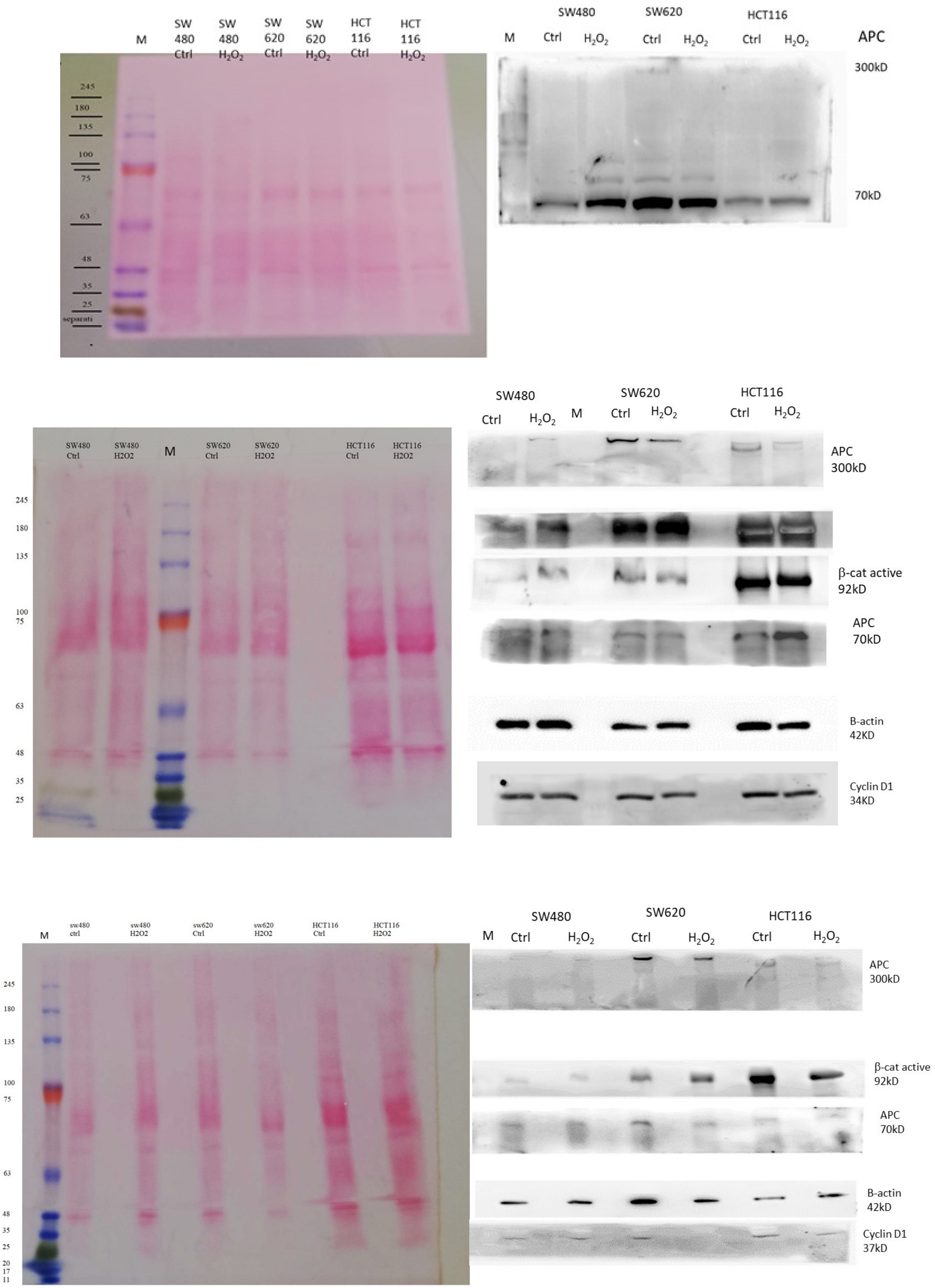

Original whole Blot.
